# Supplementary material for: Controlled gene and drug release from a liposomal delivery platform triggered by X-ray radiation
Source: Nat Commun. 2018 Jul 13;9:2713. doi: 10.1038/s41467-018-05118-3 (PMC6045614; doi:10.1038/s41467-018-05118-3)
Supplement: Supplementary file 1 — Supplementary information [file 41467_2018_5118_MOESM1_ESM.pdf]

Supplementary Information

Controlled gene and drug release from a liposomal delivery platform triggered by X-ray radiation

Deng W, et al

Supplementary Note 1: Characterization of liposome nanocomposites

Supplementary Fig.1a illustrates typical TEM images of liposomes containing gold nanoparticles and VP. Gold nanoparticle clusters were easily observed due to the higher electron density of metal gold compared with the pure liposomes (Supplementary Fig.1b). The average size of liposomes was about 165 nm determined by dynamic light scattering and the zeta potential was 37.3 ± 4 mV (Supplementary Fig.1c). Supplementary Fig.1d shows the absorption spectra of different liposome samples, where characteristic absorption peaks from both gold nanoparticles and VP were observed. We also estimated the encapsulation efficiency of oligonucleotide, Dox and epotoside loaded inside of liposomes, found to be approximately 37.5%, 44% and 77%, respectively.

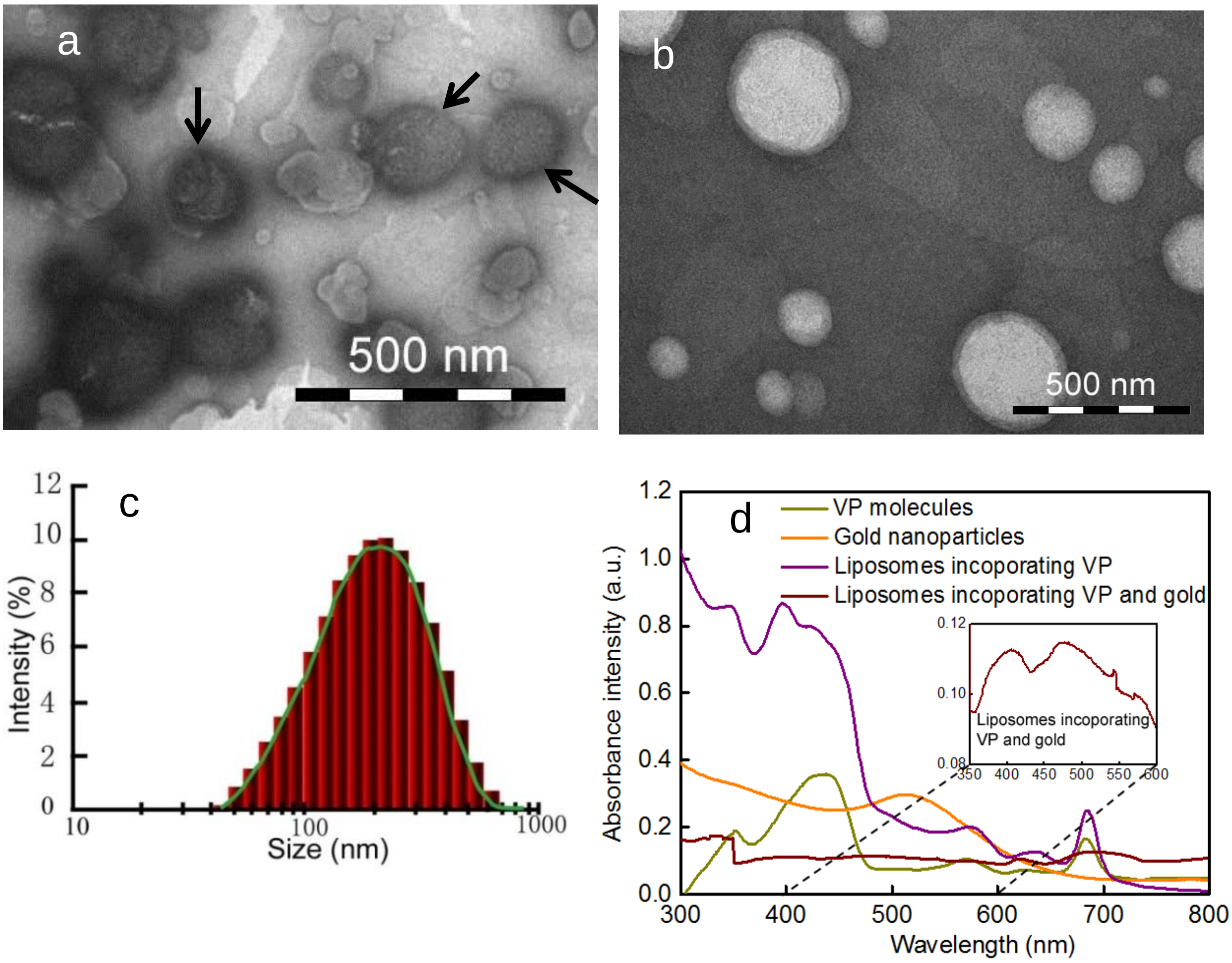

Supplementary Figure 1 Characterisation of liposomes incorporating VP and gold nanoparticles. a and b TEM images of liposomes incorporating gold nanoparticles and pure

liposomes. Black arrows indicated gold nanoparticles loaded inside liposomes. c Size distribution determined by dynamic light scattering. d Absorption spectra of liposomes, pure VP and pure gold nanoparticles. The inset shows an enlargement of absorption spectrum of liposomes incorporating VP and gold between 350-600 nm.

Supplementary Note 2: Determination of Singlet oxygen quantum yield from liposomes loaded with VP and gold nanoparticles after illumination at 365 nm wavelength

The singlet oxygen quantum yield (φ) is the ratio of the number of photons absorbed by a photosensitizer (PS) molecule to the number of singlet oxygen generated. The reference method is the most commonly used approach for calculating φ [1]. The singlet oxygen quantum yield of a PS (φ_{PS}) can be calculated based on a reference PS with a known quantum yield (φ_{REF}) using the equation [2]:

$$\varphi_{PS} = \varphi_{REF} \frac{\frac{r_{PS}}{(1-T_{PS})}}{\frac{r_{REF}}{(1-T_{REF})}} \qquad 1$$

where r_{PS} and r_{REF} are the reaction rates of the fluorescent detection probe with singlet oxygen generated from PS and reference PS respectively. T_{PS} and T_{REF} represent the transmittance of the PS and the reference PS at the illumination wavelength.

In this case, we determine the singlet oxygen quantum yield φ of liposomes loaded with VP and gold nanoparticles at 365 nm by taking φ of VP alone as the reference PS (0.53±0.06) [2]. Supplementary Figure 2a shows the variation of SOSG intensity at 525nm as a function of UV illumination time for liposomes loaded VP alone and liposomes loaded with VP and gold nanoparticles. Their absorption spectra of these nanocomposites are shown in Supplementary Figure 2b. The transmittance value at 365 nm is calculated from absorbance of VP alone and liposomes loaded with VP and gold nanoparticles based on their absorption spectra. Using the equation 1 with the reaction rate and absorbance value obtained from Supplementary Fig.2, the singlet oxygen quantum yield φ of liposomes loaded with VP and gold nanoparticles obtained in this work was estimated as 0.75±0.18. This result shows that there is an enhancement in the quantum yield value of liposomes loaded with VP and gold nanoparticles by a factor of 1.42 compared with liposomes loaded with VP alone. This enhancement is

tentatively attributed to the electric field enhancement around the gold nanoparticles present in gold-loaded liposomes.

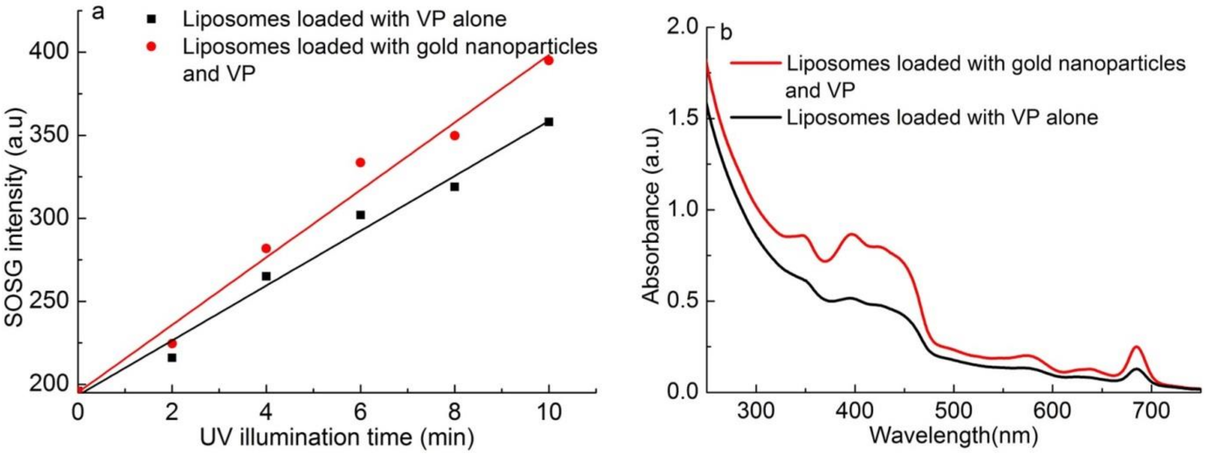

Supplementary Figure 2 Quantification of $^{1}O_2$ generation under UV illumination for liposomes loaded with VP and gold nanoparticles and liposomes loaded with VP alone. a SOSG intensity as a function of UV illumination time. b Absorption spectra of these samples.

Supplementary Note 3: Quantification of singlet oxygen from liposomes loaded with VP and gold nanoparticles under X-ray radiation

To quantify the number of singlet oxygen generated from liposomes loaded with VP and gold nanopaticles under X-ray radiation for a particular dose, we established a relation between the number of singlet oxygen molecules generated by X-ray radiation and the intensity of SOSG fluorescence, in a way similar to our previous publication [3].

We first calculated the number of UV photons absorbed ($N_{uv}(t)$) by liposomes loaded with VP alone as a function of time using the equation:

$$N_{uv}(t) = \frac{P}{E} * F * t \qquad 2$$

where P is the optical power detected on the surface of the sample, E is the energy of 365 nm photons and t is the time of illumination. F is the absorption factor and is calculated from the absorption spectra of the sample. This $N_{uv}(t)$ is plotted against time as shown in Supplementary Fig. 3a. From the known singlet oxygen quantum yield φ of VP mentioned above and $N_{uv}(t)$ from Supplementary Fig. 3a, we calculated the number of singlet oxygen generated corresponding to each UV photons absorbed. If we compare this number with the SOSG intensity in Supplementary Fig. 2a, we will obtain a conversion factor which gives the calibration of the SOSG signal with respect to the number of singlet oxygen generated.

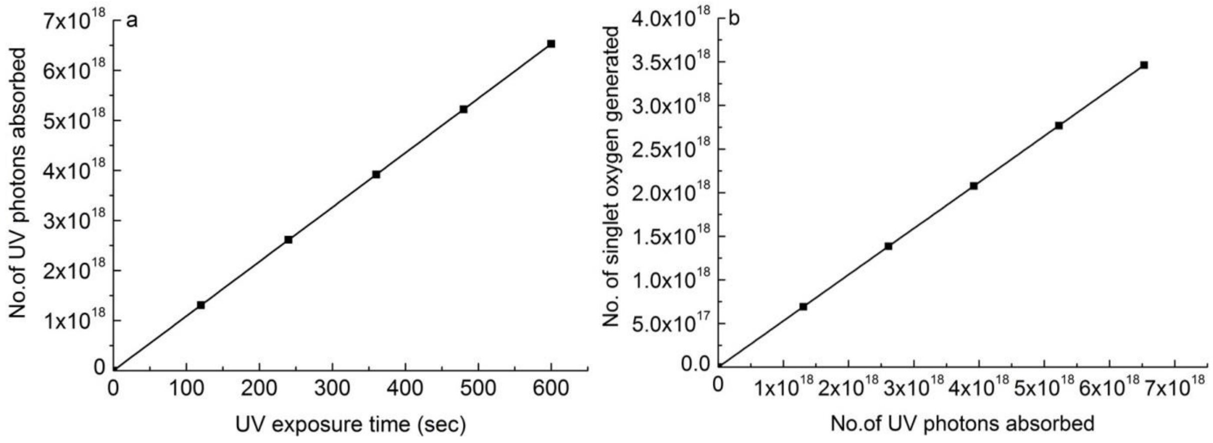

Supplementary Figure 3 Quantification of singlet oxygen from liposomes under light illumination. a Number of UV photons absorbed by liposomes loaded with VP alone as a function of time. b Number of singlet oxygen generated versus number of UV photons absorbed.

Supplementary Fig. 4a shows the SOSG intensity as a function of X-ray dose applied to liposomes loaded with VP and gold nanoparticles. By using the conversion factor estimated above, we now calculate the number of singlet oxygen generated corresponding to each X-ray dose. In this case, the number of singlet oxygen generated from liposomes loaded with VP and gold nanoparticles for 4 Gy is ~ 2.9×10^{18}. By dividing the number of liposomes in this sample, we estimate the number of singlet oxygen generated from each liposome, which is about 7250. In this case, we took into account of the fact that SOSG shows some background fluorescence due to the presence of endoperoxides generated before the exposure to X-ray radiation.

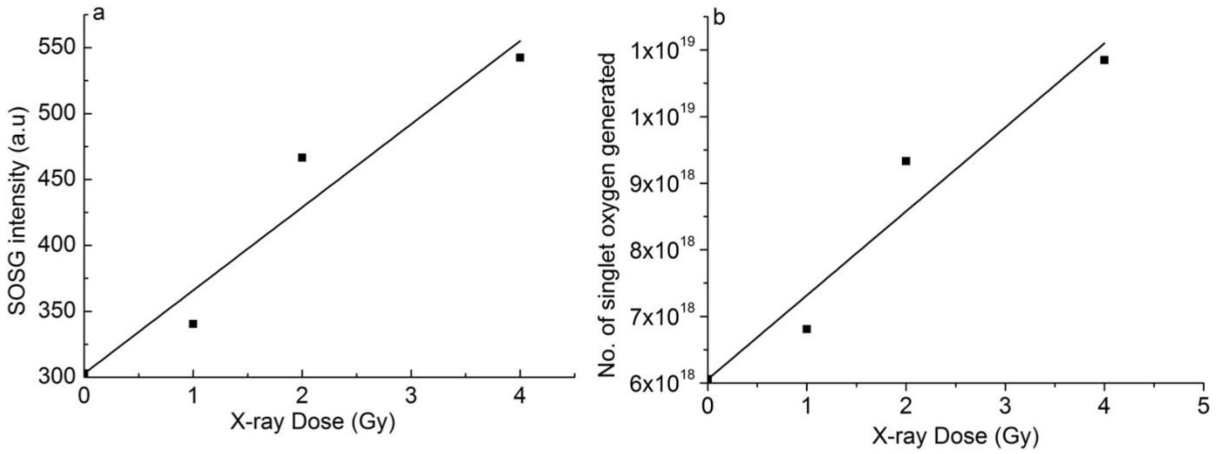

Supplementary Figure 4 Quantification of singlet oxygen from liposomes under X-ray radiation. a SOSG intensity as a function of X-ray Dose for liposomes loaded with VP and gold nanoparticles. b Number of singlet oxygen generated corresponding to each X-ray dosage.

Supplementary Note 4: Calculation of the number of liposomes per cell and the number of gold nanoparticles per liposome

We first calculate the number of lipid molecules in each liposome as per the equation [4]:

$$N_{tot} = \frac{\left[4\pi(\frac{d}{2})^2 + 4\pi[\frac{d}{2} - h]^2\right]}{a} \qquad 3$$

where d is the diameter of a liposome, h indicates the thickness of a liposomal bilayer that was calculated as 4.7 nm for our lipid formulation [5], and a represents the average lipid head group area, whose value is calculated according to $a = a_1 N_1 + a_2 N_2 + a_3 N_3 + \cdots$, where N is the molar fraction of each lipid component and a is 70Å for DOTAP [6] and 72.4 Å for DOPC [7] in our study.

The number of liposome for a known concentration of lipids is estimated by using the equation:

$$N_{lipo} = \frac{[lipid]\times N_A}{N_{tot}\times 1000} \qquad 4$$

where $[lipid]$ is the lipid concentration, N_A is the Avogadro number (6.023×10^{23} mol L^{-1}) and N_{tot} is the total number of lipids per liposome.

The cell number is counted before incubation with liposomes. The total number of liposome is calculated based on Equation 3 and 4 after cell lysis and average number of liposomes taken up by each cell is obtained by dividing the total number of liposomes with the cell number.

For the number of gold nanoparticles per liposome, we first calculate the total number of gold atom (N_{atom}) in our liposome sample based on ICP-MS analysis and equation 5:

$$N_{atom} = \frac{[Au^{3+}]\times V}{M} \times N_A \qquad 5$$

where $[Au^{3+}]$ is the concentration of Au (III), V stands for the sample volume, M indicates the atomic weight of gold and N_A is the Avogadro number (6.023×10^{23} mol L^{-1}).

The average number of gold atoms per gold nanoparticle (U) is also calculated by using the following equation [8]:

$$U = \frac{2}{3} \times \pi \times (\frac{D}{\alpha})^3 \qquad 6$$

Where D refers to the diameter of gold nanoparticle and α is the edge of a unit cell whose value was 4.0786 Å. Therefore the number of gold nanoparticles (N_{gold}) in a liposome sample is calculated based on the equation:

$$N_{gold} = \frac{N_{atom}}{U} \qquad 7$$

Finally the number of gold nanoparticles per liposome (N) is estimated as per the equation:

$$N = \frac{N_{gold}}{N_{lipo}} \qquad 8$$

Supplementary Note 5: Serum and pH stability studies of PEGylated liposomes

For serum stability studies, the cumulative percentage of Dox released from liposomes with and without PEG modification is shown in Supplementary Fig.5a and 5b. Different amounts of Dox were released from conventional liposomes during 48 hr incubation, with the total amount being more than 30% and 50% at 48 hr when incubated in PBS with 10% and 50% FBS (Supplementary Fig.5a). However, the Dox release profile shown in Supplementary Fig.5b indicated that the release rates were reduced in the PEGylated liposomes, compared with liposomes without PEGylation. Liposomes still retained more than 90% and 80% of their initial drug content at 48 hr incubated in PBS with 10% and 50% FBS, indicating that PEG chains on the liposome surface would contribute to improved its stability in the blood circulation. Considering that decreased pH is a major feature of tumour tissue and, in principle, it may affect liposome stability and drug release from the liposomes. We also assessed Dox release triggered by different values of pH (Supplementary Fig.5c). These PEGylated liposomes showed a similar Dox release profile at different buffer pH values (7.4, 6.0 and 5.0). The overall amount of released Dox was less than 10% for 48 hr incubation even at pH 5.0. These findings suggested that the liposome formulation prepared in this study was largely unaffected by the decreased pH value. This indicates no stability change of liposomes in the tumour microenvironment before the application of light or X-ray to the tumour site.

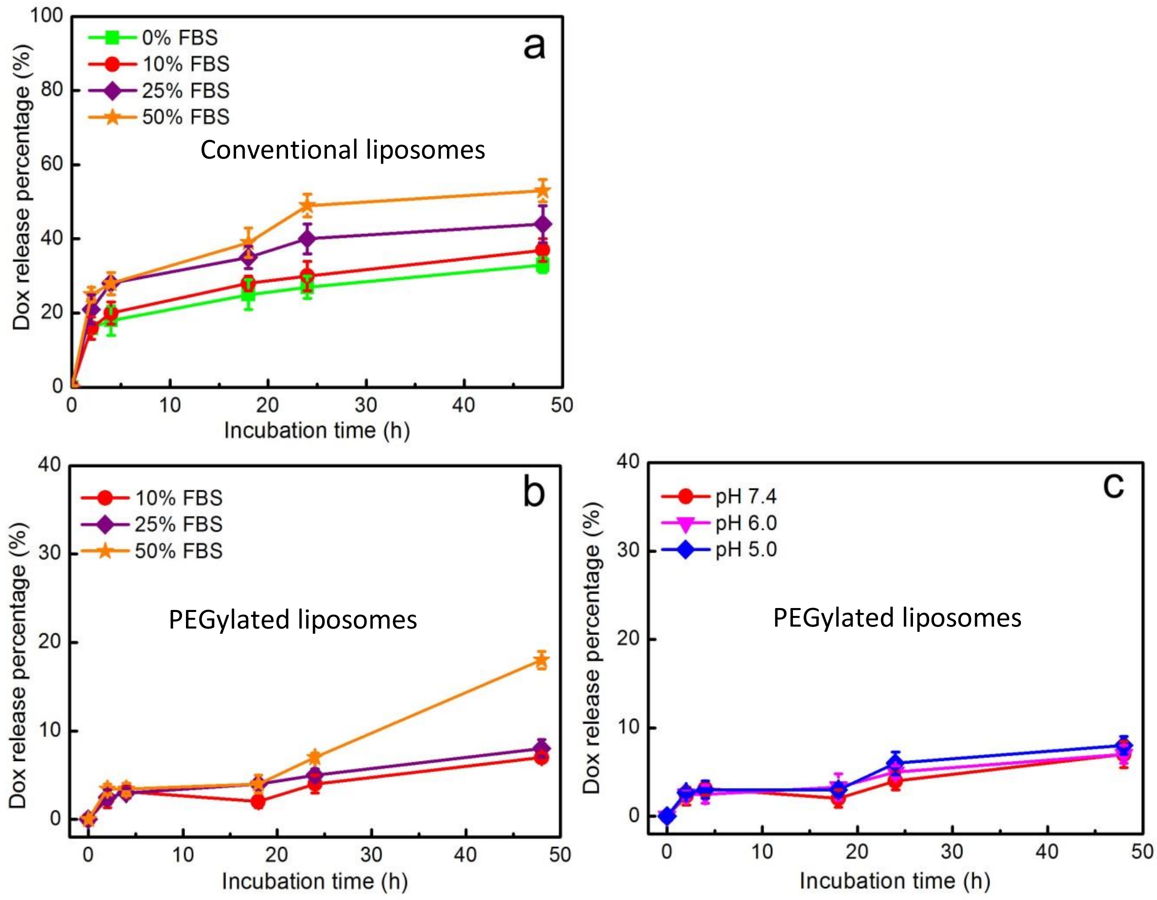

Supplementary Figure 5 Serum and pH stability studies of PEGylated liposomes. a-b The percentage of released Dox from (a) conventional liposomes and (b) PEGylated liposomes after 0 hr, 2 hr, 4 hr, 18 hr, 24 hr and 48 hr incubation in PBS (pH 7.4) containing FBS with various concentrations. c The percentage of released Dox from PEGylated liposome samples incubated in PBS (pH 7.4, 6.0 and 5.0) containing 10% FBS. Error bars show standard deviation from three measurements.

Supplementary Figure

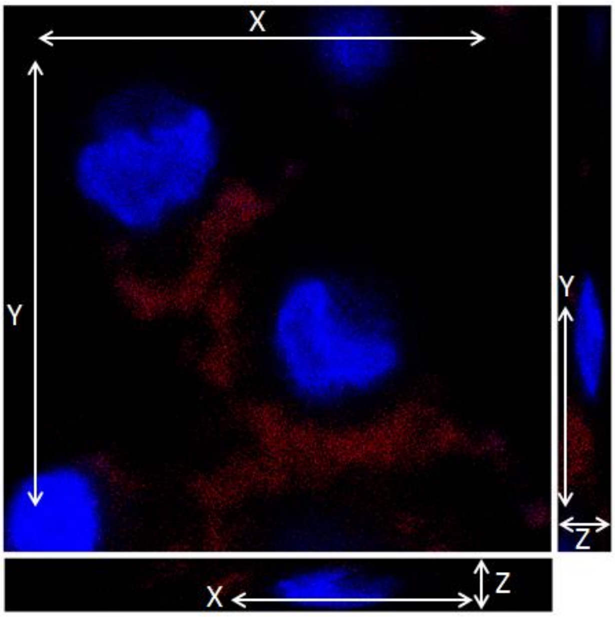

Supplementary Figure 6 Orthogonal views (XY, XZ and YZ) of cellular uptake of liposomes loaded with verteporfin after 4-h incubation with PC12 cells. The X-Y plane image was obtained at the centre of the Z-stack.

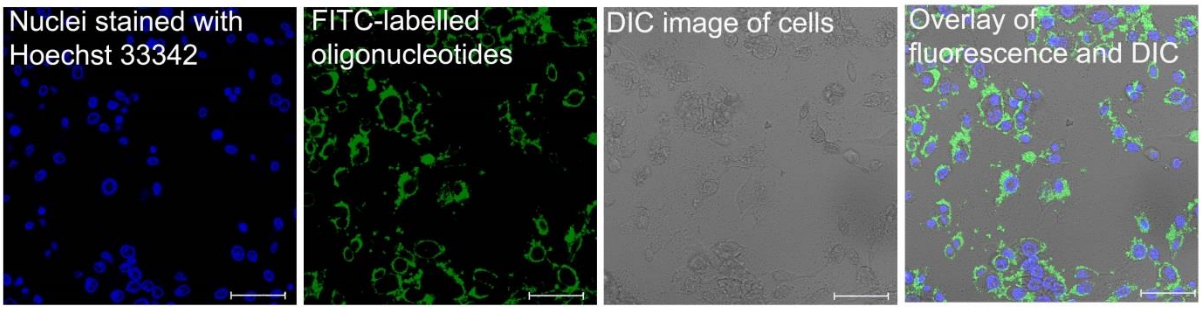

Supplementary Figure 7 Confocal laser scanning microscopy images of PC12 cells incubated with liposome nanoparticles (25 µM) loaded with fluorescent oligonucleotides for 4 hr. Scale bar is 75 µm.

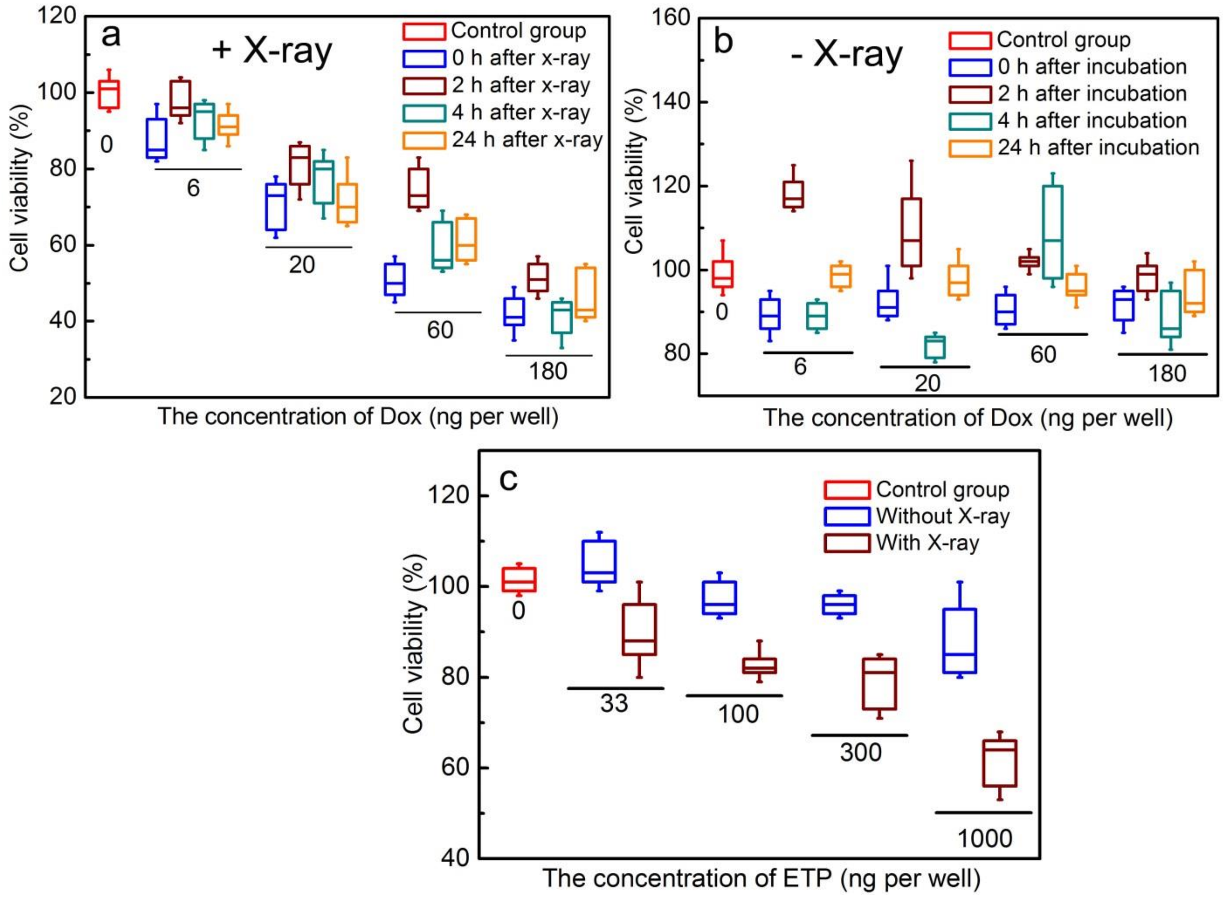

Supplementary Figure 8 X-ray triggered *in vitro* chemotherapy. a-b Cell-killing effect of LipoDox on HCT 116 (a) with and (b) without X-ray radiation of 4 Gy at various time points (0 hr, 2 hr, 4 hr and 24 hr). The concentration of Dox was 6, 20, 60 and 180 ng per well. c Cell-killing effect of LipoETP on HCT 116 at 24 hours after X-ray radiation of 4 Gy. The concentration of ETP was 33, 100, 300 and 900 ng per well. The box is bounded by the first and third quartile with a horizontal line at the median and whiskers extend to 1.5 times the interquartile range.

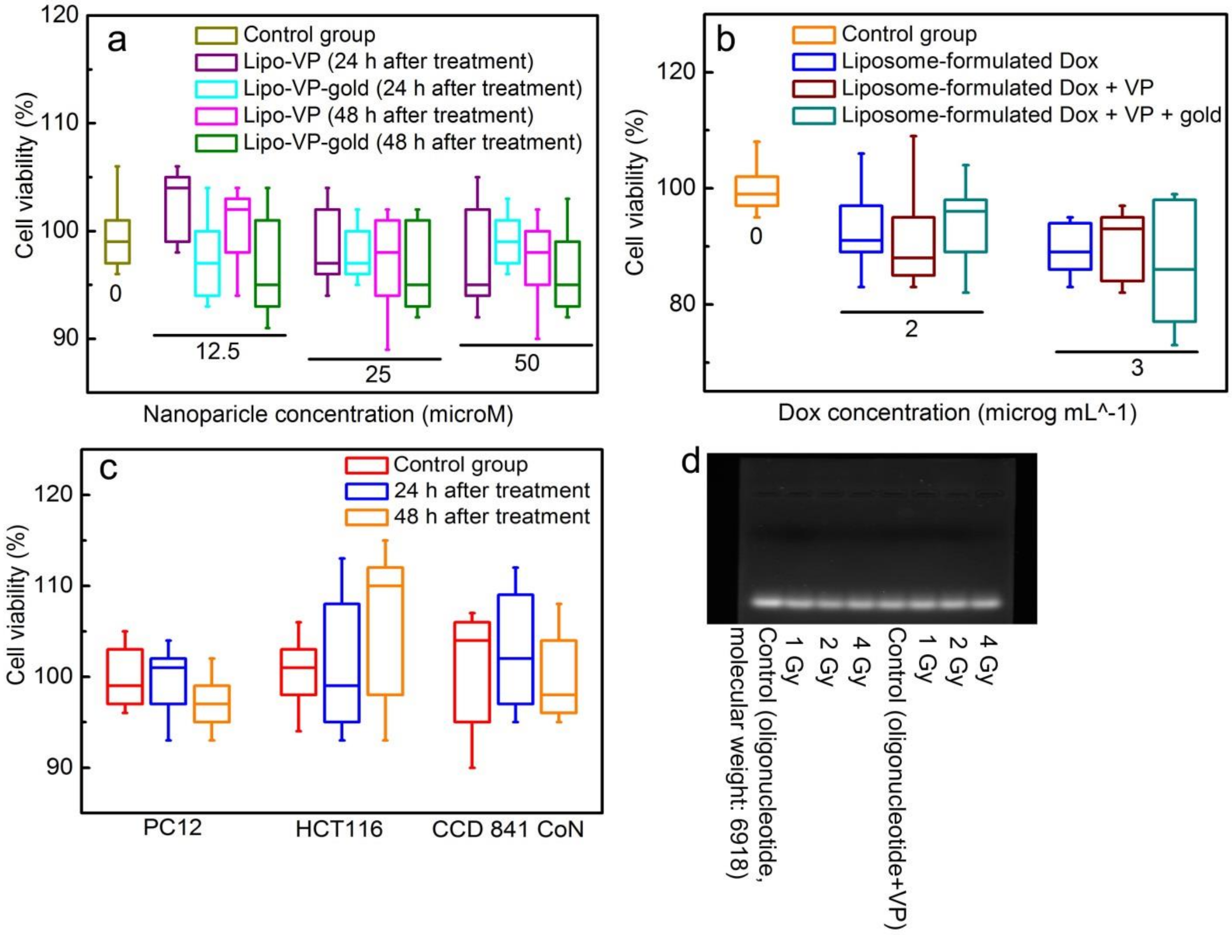

Supplementary Figure 9 *In vitro* toxicity assessment of liposome samples and X-ray radiation on cells and oligonucleotide. a Cell viability of PC12 cells at 24 hr and 48 hr after treatment with liposomes incorporating VP (Lipo-VP) and gold nanoparticles (Lipo-VP-gold). b Viability of CCD 841 CoN cells at 24 hr after treatment with liposome-formulated Dox. c Toxicity of X-ray of 4 Gy on PC12, HCT 116 and CCD 841 CoN cells at 24 hr and 48 hr after treatment. The box is bounded by the first and third quartile with a horizontal line at the median and whiskers extend to 1.5 times the interquartile range. d Agarose gel electrophoreses of antisense oligonucleotide (10 μg mL^{-1}) and mixture of oligonucleotide and VP (10 μg mL^{-1}oligonucleotide and 32 μg mL^{-1}verteporfin) after X-ray exposure with different dosage. From left to right lane: control sample without treatment, 1 Gy, 2 Gy and 4 Gy.

Supplementary Note 6: Histological analysis of tumour tissues after each treatment

Supplementary Fig.10a demonstrates the histological images of tumour tissue under different treatment conditions. In the PBS-treated group, the internal region of the tumours mainly consisted of non-viable tumour residuals (about 1/3 of the whole volume of the lesion), while the outer part was formed by viable tumour cells. In the LipoDox-treated group, the viable tumour tissue formed a thinner outer rim (up to 0.5 mm below the capsule), but, in contrast to the PBS-treated group, it protruded towards the inner region as elongated cords and alternated with the necrotic and paranecrotic sites. The volume of non-viable tumour tissue in LipoDox group was about 60-70%. In the X-ray treated group, the difference between the outer and inner regions was less pronounced compared with other groups, and bigger fragments of viable tumour tissue of solid structure were surrounded by non-viable tumour tissue residuals. Finally, in the group treated by X-ray triggered LipoDox, the structure of the tumour resembles that observed in the LipoDox group, but with a significant reduction of the relative volume of viable tumour tissue. In particular, the outer rim of viable tumour tissue is much thinner (only 100-300 μm in thickness), and the amount of the viable tumour tissue spreading into the internal part of the tumour is significantly less than in any other experimental groups. In contrast to all other groups, the necrotic tumour tissue was visible even in the subcapsular zone. The average volume of necrotic tissue in the tumours was about more than 80%.

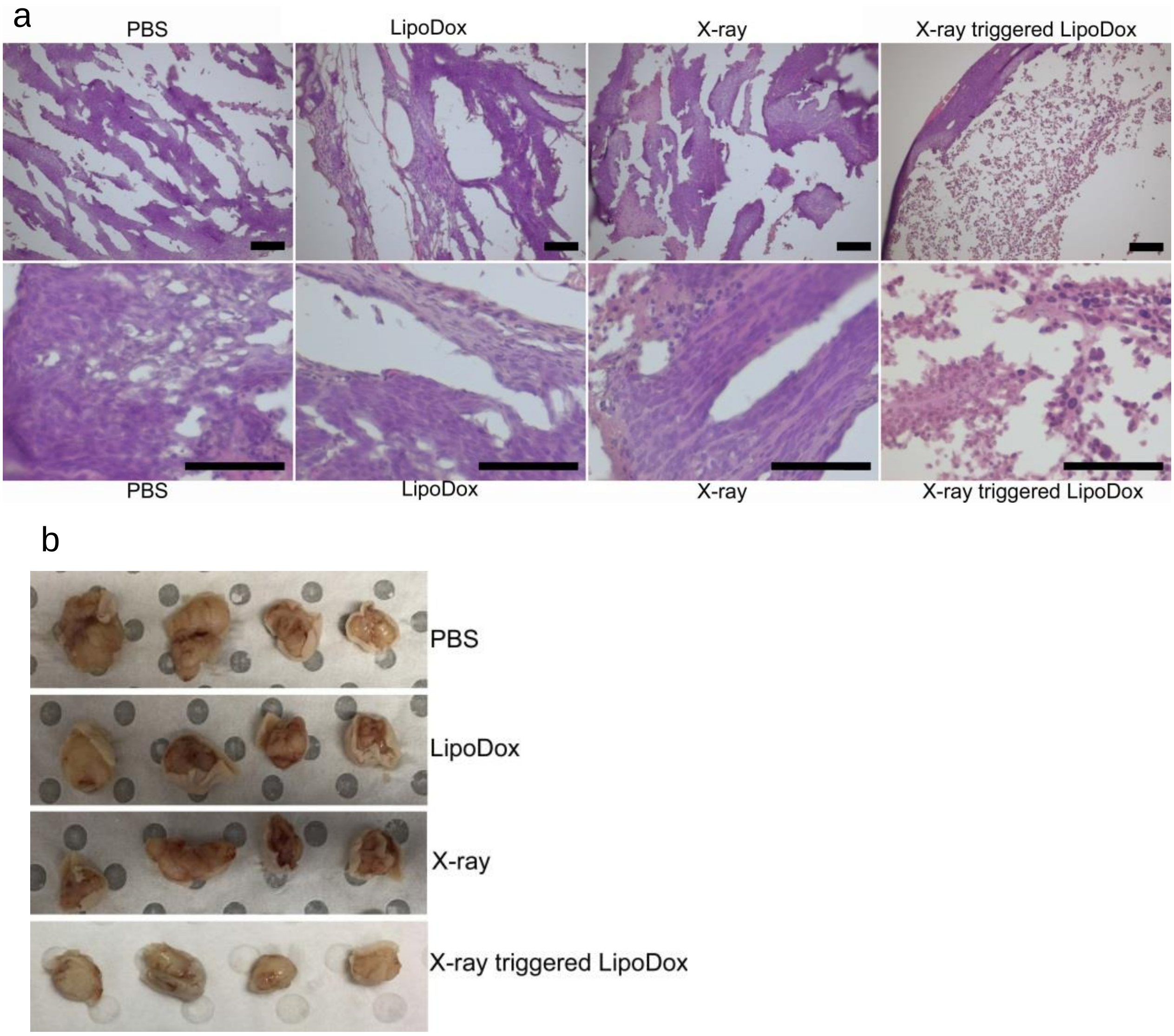

Supplementary Figure 10 *In vivo* antitumour effect by X-ray triggered LipoDox. a Representative histological images of tumour tissues after various treatments. H & E staining. The scale bar is 100 µm. b Photographs of tumours isolated at the endpoint.

Supplementary references:

1. Lin, H. et al. Feasibility study on quantitative measurements of singlet oxygen generation using singlet oxygen sensor green. *Journal of fluorescence* **23**, 41-47 (2013).
2. Clement, S., Sobhan, M., Deng, W., Camilleri, E. & Goldys, E.M. Nanoparticle-mediated singlet oxygen generation from photosensitizers. *Journal of Photochemistry and Photobiology A: Chemistry* (2016).
3. Clement, S., Deng, W., Camilleri, E., Wilson, B.C. & Goldys, E.M. X-ray induced singlet oxygen generation by nanoparticle-photosensitizer conjugates for photodynamic therapy: determination of singlet oxygen quantum yield. *Scientific reports* **6** (2016).
4. Güven, A., Ortiz, M., Constanti, M. & O'Sullivan, C.K. Rapid and efficient method for the size separation of homogeneous fluorescein-encapsulating liposomes. *Journal of liposome research* **19**, 148-154 (2009).
5. Small, D.M. Lateral chain packing in lipids and membranes. *Journal of Lipid Research* **25**, 1490-1500 (1984).
6. Koltover, I., Salditt, T. & Safinya, C. Phase diagram, stability, and overcharging of lamellar cationic lipid–DNA self-assembled complexes. *Biophysical Journal* **77**, 915-924 (1999).
7. Kučerka, N., Tristram-Nagle, S. & Nagle, J.F. Structure of fully hydrated fluid phase lipid bilayers with monounsaturated chains. *The Journal of membrane biology* **208**, 193-202 (2006).
8. Chithrani, B.D., Ghazani, A.A. & Chan, W.C. Determining the size and shape dependence of gold nanoparticle uptake into mammalian cells. *Nano letters* **6**, 662-668 (2006).
